# The effect of outcome-based education on clinical performance and perception of pediatric care of the third-year nursing students in Mongolia

**DOI:** 10.1371/journal.pone.0305298

**Published:** 2024-06-11

**Authors:** Khishigdelger Lkhagvaa, Basbish Tsogbadrakh, Gankhuyag Gochoosuren, Oyungoo Badamdorj, Azadeh Stark

**Affiliations:** 1 School of Nursing, Mongolian National University of Medical Sciences, Ulaanbaatar, Mongolia; 2 School of Interdisciplinary Studies, University of Texas at Dallas, Richardson, Texas, United States of America; 3 Department of Pathology & Laboratory Medicine, Henry Ford Health, Detroit, Michigan, United States of America; Al-Ahliyya Amman University, JORDAN

## Abstract

**Background:**

Mongolian government has set improvement of clinical proficiency of nursing students as one of its priorities. Nursing professionals have the sentinel role in providing healthcare services in rural areas. Outcome-based education (OBE) offers a promising pedagogical approach to actively mentally engage students to strengthen their clinical proficiencies. We implemented a pilot project with the objective of comparing students’ clinical performance under OBE with our traditional didactic techniques.

**Methods:**

The researchers implemented a non-equivalent two-armed quasi-experimental post-test-only’ design approach study. The intervention arm (n = 34) received OBE pediatric training, while the control arm (n = 32) received the traditional pedagogical pediatric nursing training. Each arm of the study completed 16 hours of theory, 32 hours of clinical skills practice and 32 hours of seminars in pediatric nursing care. Data were collected using a five-section instrument, Demographic, Competency Inventory, Nursing Students’ Satisfaction, Course Experience, and Objective Structured Clinical Examination. Performance and knowledge proficiencies were evaluated by applying the two-sided independent T-test. The distributions of categorical variables were assessed by Fisher’s exact test or chi-squared test of significance.

**Results:**

The intervention arm had higher mean score value in the competency inventory ($\bar{X}$ = 238.70, SD = ± 23.07) compared to the control arm ($\bar{X}$ = 222.11, SD = ± 39.94) (P = 0.04); similarly, the mean value for nursing students’ satisfaction was higher for the intervention arm ($\bar{X}$ = 117.87, SD = ± 15.94) compared to the control group ($\bar{X}$ = 109.76, SD = ± 16.94) (P = 0.049). Additionally, the difference in the mean value for course experience questionnaire between the intervention arm ($\bar{X}$ = 125.33, SD = ± 19.30) and the control arm ($\bar{X}$ = 110.41, SD = ± 11.28) was statistically significant (P = 0.0001). Finally, the intervention arm had a higher mean value ($\bar{X}$ = 85.40, SD = ± 6.11) for objective structural clinical examination compared to the control arm ($\bar{X}$ = 81.56, SD = ± 7.01) (P = 0.023).

**Conclusion:**

OBE pedagogical approach offers promising benefits to improving nursing students’ clinical competencies; additionally, the OBE approach seems to increase students’ satisfactions with their clinical curriculum.

## Introduction

The burden of maternal and infant morbidity and mortality (MMR), particularly in low- and middle-income countries (LMICs) has been a global concern [1]. This burden is mostly observed in resource limited regions, i.e., rural areas of LMICs [2]. For example, in Mongolia, the average annual MMR in remote aimags (provinces), depending on their geographic locations, is between 4 to 6 times higher than the capital city, Ulaanbaatar [3]. Mongolian public health policies and laws have delegated the primary role and responsibilities of the delivery of maternal and child health to the nursing professionals [4]. By virtue of this role, the nursing professionals in Mongolia carry the sentinel responsibility in closing the urban-rural MMR gap [3, 5].

Improvement of clinical dexterity of nursing students and closing the gap between theory and clinical practice is one of the objectives on the educational agenda of the Mongolian government. This objective became a priority after a relatively recent findings from an international study which ranked Mongolian nursing students at the lowest level of dexterity in clinical practice when compared with their international counterparts [6]. Academic institutions in Mongolia have been striving to re-architect their nursing curricula with the objective of narrowing the gap between theory and clinical skills of nursing students, particularly in the field of maternal health and pediatrics [7].

Pediatric Nursing is a required course for students who choose to specialize in this field. Students are expected to learn about pediatric anatomy, physiology, and psychology, in addition they are expected to learn about a spectrum of childhood diseases [8]. The volume of information covered in this course can be challenging for many students and even overwhelming for some. Under the traditional didactic approach, members of the faculty disseminate the information to the students and students are expected to be passive learners. The traditional teaching approach essentially neglects students as the main pedagogic players. Many students have difficulties with understanding and processing the information despite the efforts of members of the faculty [8].

Mongolian nursing students are faced with another limitation, which is the limited number of hours in pediatric clinics. They are required only 48 hours of pediatric clinical hours, in contrast to 61 to 80 clinical hours in other countries. The limited clinical experience deprives students of the opportunity for hands on experience and application of theory into practice [9].

The Outcome-Based Education (OBE) teaching method offers the opportunity to overcome limitations of the traditional method of education. OBE is a pedagogical theory which emphasizes designing and implementing an educational intervention around goals or outcomes. These goals or outcomes, e.g., demonstration of dexterity in practical application of knowledge, are clearly communicated with students [10, 11]. Furthermore, OBE is effective in capturing and maintaining the interest and attention of students and fostering their critical thinking [12, 13].

The Mongolian “Gen Z”, the generation born between 1994 and 2012, similar to their cohort across the globe, rate the uniqueness of the delivery of educational messages and the novelty of training methods as the two most important characteristics of an effective teaching and an educational program [14]. Academic nursing professionals in several countries in response to the “cultural values” of the “Gen Z” generation have embraced the notion of outcome-based education (OBE) and have re-architected their didactic strategies and teaching practices [10, 15, 16].

The call for transformation of nursing curriculum and narrowing the gap between theory and practice has been reverberated in the nursing literature and has been emphasized by Mongolian government [7, 17–19]. The current findings suggest the potential benefits of OBE; however, there is a paucity of research about the value of OBE from students’ perspective; furthermore, so far, no study has been implemented in Mongolia to assess the effectiveness of OBE pedagogy among nursing students. Therefore, we implemented a pilot study to test the hypothesis that the OBE pedagogical approach offers the nursing students a better learning experience during their pediatric nursing course. This better learning experience would then be reflected as an improved level of satisfaction with the course and better assessment abilities and application of theoretical knowledge into practice. The specific aims of our study were: 1) To discern and compare the level of students’ satisfaction under the OBE method of teaching with the traditional method of teaching the pediatric nursing course. 2) To assess and compare students’ abilities in application of theoretical knowledge into practice taught under the OBE method with the traditional method.

## Methods

### Setting

The School of Nursing, Mongolian National University of Medical Sciences (MNUMS) was founded in 1929 in Ulaanbaatar by the decision of the government of Mongolia. The school is the first academic institution of higher education in nursing sciences. Since opening its doors to the first class of nursing students about a century ago, more than 11,000 students have graduated from the school. Currently, the school is 80 faculty members and 1,348 students strong. The accredited nursing program successfully passed the criteria by the Accreditation Agency for Study Programmes of Engineering, Information Science, Natural Sciences and Mathematics (ASIIN). The ASIIN accreditation is a seal of approval for institutional high standards for quality management and comprehensive competencies in professional training and continuous self-evaluation [20].

### Study design and scope

We implemented a non-equivalent two-armed quasi-experimental post-test-only’ design approach study [21, 22].

The intervention arm (n = 34) received the outcome-based pediatric nursing pedagogical training, while the control arm (n = 32) received the traditional pedagogical pediatric nursing training. Participants in both arms of the study completed, in parallel, a total of 16 hours of theory, 32 hours of clinical skills practice and 32 hours of seminars in pediatric nursing care during the second semester of the 2021–2022 academic year at the School of Nursing, MNUMS. The training objectives, delivery strategies and training outcome assessment methods are presented in Table 1. Study participants completed a total of four modules, with training for each module lasting for three weeks. Therefore, the duration of training for participants in either arm of the study was a total of 12 weeks. The training sessions for the two arms of the study were conducted in parallel. At the end of each module, the performance of the study participants were evaluated by the qualified members of the faculty at the School of Nursing, MNUMS, according to the standardized evaluation protocols. These protocols were developed to ascertain uniformity and consistency in evaluation of the participants by the members of the faculty. At the end of the 12 weeks of training, the study participants were asked to complete self-administered questionnaires. We have provided a detailed explanation about the design and structure of this self-administered questionnaire under the sub-heading of Research Instrument.

**Table 1:** Training objectives, delivery strategies, and training outcome assessment methods for the intervention and the control arms of the study.

Item	Intervention	Control group
Theory based intervention	Outcome-based education Outcome based course design using concept mapping.	Objective based curriculum design Teaching-traditional training
Delivery	Instructors of pediatric nursing with 7 and 31 years of clinical experience in the pediatric nursing.	Instructors of pediatric nursing with 7 and 31 years of clinical experience in pediatric nursing.
Hours	16 hours theory, 32 hours clinical skills practice and 32 hours seminar.	16 hours theory, 32 hours clinical skills practice and 32 hours seminar.
Learning objectives	• Understand anatomy, physiology, and pathology of pediatric disorders systems. • Analyze results of common laboratories and examinations on pediatric disorders. • Discriminate results of pediatric disorders functional assessment. • Analyze treatment side effects and interventions of common pediatric diseases. • Comprehend common pediatric nursing problems and nursing priorities.	• Identify anatomy, physiology, and pathology of pediatric disorders systems. • Apply assessment skills of the pediatric disorders system to collect history and assess pediatric disorders functions. • Demonstrate the purposes of nursing care before and after examination of pediatric disorders. • Comprehend etiologies, clinical symptoms, and treatments of common diseases of pediatric disorders. • Demonstrate common pediatric nursing problems and nursing priorities.
Teaching strategies	Lecture Group discussion Concept demonstration Concept mapping construction (5–6 students per group) Performance-based skills	Lecture Group discussion Concept demonstration Problem based learning
Formative evaluation	Test of knowledge of pediatric nursing and case discussion. Rubrics evaluation Concept mapping of scenario-based nursing care plan. OSCE (Objective Structured Clinical Examination)	Test of knowledge of pediatric nursing and case discussion. OSCE (Objective Structured Clinical Examination)
Summative evaluation	Nursing competency Course learning outcome	

### Study participants

#### Population and sample

The cohort of third-year nursing students (n = 137) were recruited via the university email system to participate in our study. A total of five classes, each 26 to 28 members strong, constitutes this cohort of 137 nursing students. In the recruitment emails, we introduced the objective and the scope of the study. A total of 66 students volunteered to contribute to our study. The minimum number (n = 66) was calculated based on a power of 0.80 (β = .8), probability of type I error of 0.05 (α = 0.05) and an estimated effect size of 0.71; this estimated effect size was based on a previously published study [23]. The control arm was comprised of 32 participants, while a total of 34 participants constituted the intervention arm.

#### Eligibility criteria

Students were eligible to participate in our study if: if 1) they were the third-year students in the nursing baccalaureate degree program. 2) If they were enrolled in the pediatric care nursing course during the second semester of 2021–2022 academic year at the School of Nursing, MNUMS and 3) If they were willing to participate in our study. Therefore, students who did not meet the three eligibility criteria were excluded from the study.

#### Research instrument

We designed and developed a five-section instrument to collect data on demographic variables, nursing competencies, course experience, course satisfaction and assessment of theoretical knowledge and clinical proficiency of the participants.

*Demographic Section I*. The demographic section which is the first section (Section I) of our instrument was designed with the objective of capturing data on age, gender, grade point average and residential region (rural or urban setting). This section contains a total of 4 questions.

*Competency Inventory of Nursing Students (CINS) Section II*. The objective of section II of our instrument, the Competency Inventory of Nursing Students (CINS), was to capture data on students’ competency in clinical nursing knowledge and skills. CINS was grounded in the work of Hsu and Hsieh (2013) [24]. This section of the instrument, which was designed to assess competency in clinical nursing knowledge and skills, is comprised of 6 subscales, accounting for a total of 43 items. Each item is scaled on a seven-point Likert scale. The overall possible score for this section can range from a low of 43 to a high of 301 points. Scores were trichotomized into three categories of low (0–100), moderate (101–200), and high (201–301) levels of competency [24]. The higher score indicates higher competency in clinical nursing knowledge and skills. The Cronbach’s alpha coefficient of internal consistency and reliability for CINS was 0.95.

*Nursing Students’ Satisfaction Scale (NSSS) Section III*. The Nursing Students’ Satisfaction Scale (NSSS) forms section III of our instrument. We developed NSSS, based on the work of Chen et al. with the objective of capturing data on students’ satisfaction about OBE or traditional didactic method (2012) [25]. The NSSS consists of 27 items and each item is scaled on a six-point Likert scale ranging from 1 (not satisfied at all) to 6 (very satisfied). The overall possible score for this section can range from a low of 27 to a high of 162 points. Scores were trichotomized into three categories of low (0–54), moderate (55–108), and high (109–162) levels of satisfaction [25]. A higher score indicates a higher level of satisfaction. The Cronbach’s alpha coefficient of internal consistency and reliability for NSSS was 0.93.

*Course Experience Questionnaire (CEQ) Section IV*. Course Experience Questionnaire (CEQ) constitutes section IV of our instrument. This section was designed with the objective of collecting data on students’ perceptions about their experiences with either OBE pedagogy or the traditional method of teaching. We benefitted from the work by P. Ramsden (1991) to design and develop the CEQ segment or the fourth segment of our instrument [26]. This segment contains 6 dimensions, accounting for a total of 36 items. Each item is scaled on a 5-point Likert scale, ranging from 1 (Definitely Disagree) to 5 (Definitely Agree). The coding of the 15 negatively worded items was reversed so that higher scores corresponded to more positive ratings. The overall possible score for this section can range from a low of 36 to a high of 180 points. A higher score indicates a better experience with the course and the learning process. The total score of the whole scale was interpreted as low (0–60), moderate (61–120), and high (120–180) levels of satisfaction [26]. The Cronbach’s alpha coefficient of internal consistency and reliability for CEQ was 0.83.

*Comprehensive Theoretical Knowledge and an Objective Structured Clinical Examination (OSCE) Section V*. Section V of our instrument, the Comprehensive Theoretical Knowledge, and an Objective Structured Clinical Examination Section, was designed and developed with the objective of capturing data on students’ clinical performance and their dexterity in clinical application of theoretical knowledge. This segment of our instrument was architected based on the required standard nursing curriculum; furthermore, OSCE is the widely applied method for evaluation of clinical dexterity of nursing student in Mongolia [27]. This section is comprised of 30 items. The possible maximum grade is 100, while the score of 60 is considered as the minimum acceptable passing score. The Cronbach’s alpha coefficient of internal consistency and reliability for OSCE was 0.85.

### Ethical considerations

This study was approved by the Ethic Board of the Mongolian National University of Medical Sciences (#2022/01-21, 2022/3-01). All participants were informed about the objective and method of the study. Additionally, they were free to refuse to participate or withdraw from the study at any time with no academic repercussions. All participants signed informed consent forms before participating in the study. Finally, participants’ confidentiality was strictly protected. Study participants were identified by unique study numeric identifiers. Links to the names and the other personal identifiers were destroyed after the last step of data quality and data assurance.

### Statistical analysis

We used descriptive statistics to summarize the demographic characteristics of the study participants and the outcomes of CEQ, NSSS, CINS, comprehensive theoretical knowledge and OSCE assessments. We then applied the two-sided statistical method of independent T-test to assess and compare the distribution of continuous variables between the two arms of the study. The distributions of categorical variables were assessed by Fisher’s exact test or chi-squared test of significance. The statistical significance value was set at 0.05 (P = 0.05). Statistical analysis was performed with SPSS Version 21.0 statistic software package.

## Results

All participants (n = 66) in both arms of the study completed the entire training period which lasted for 12 weeks. The mean age of the study participants in the control arm of the study was 22.75±0.76; while the mean age for the intervention arm was 22.56±0.68 (P = .305). Female students constituted the majority in either arm of the study, 92.62% in the control arm vs. 94.12% in the intervention arm. Participants in the control arm of the study had the mean grade point average (GPA) of 3.38± 0.20 out of the scale of 4.0; the average GPA for the participants in the intervention arm was 3.36±0.28 (P = 0.513). Urban dwellers constituted 62.5% (n = 20) of the participants in the control arm of the study and 52.9% (n = 18) in the intervention arm of the study (P = 0.432). (Table 2)

**Table 2:** Demographic characteristics of the participants in the control and intervention arms of the study (n = 66).

Variables	Control Arm (n = 32)	Intervention Arm (n = 34)
Age (years)	22.75±0.76	22.56±0.68
Gender		
Female	29 (90.62)	32 (94.12)
Male	3 (9.38)	2 (5.88)
GPA^1^	3.38±0.20	3.36±0.28
Regions		
Urban	20 (62.5)	18 (52.94)
Rural	12 (37.5)	16 (47.06)

^1^GPA: Grade Point Average

In Table 3, we have tabulated the mean values (± SD) for each dimension and the overall mean values for the three variables CEQ, NSSS and CINS for the control and the intervention arms of the study. Additionally, we have tabulated the rankings for each arm of the study based on their calculated values for each of the three variables, CEQ, NSSS and CINS. For the variable CEQ, the mean score value for the participants in the control arm of the study was calculated at ($\bar{X}$ = 110.41±11.28, range = 92–133), which corresponds with the moderate category. Meanwhile, the calculated mean score value of CEQ was ($\bar{X}$ = 125.33 ±19.30, range = 103–151) (P = 0.0001) for the participants in the intervention arm of the study. This value corresponds to the high category. For the variable NSSS, participants in the intervention arm of the study had a higher mean score value (117.87±15.94, Range = 94–162) compared to the control group ($\bar{X}$ = 109.76 ±16.94, Range = 86–147) (P = 0.049). Meanwhile, both arms were categorized into the high level of satisfaction based on their mean value scores. For the variable CINS, results of our analysis categorized participants in either arm of the study into the high level of competency; however, the mean score value ($\bar{X}$ = 238.70±23.07, Range = 199–289) for the participants in the intervention arm of the study was higher than the mean score value ($\bar{X}$ = 222.11± 39.94, Range = 178–285); this difference reached the level of statistical significance (P = 0.04). Finally, for the variable OSCE, the calculated mean value score was higher for the intervention arm of the study ($\bar{X}$ = 85.40, SD = ± 6.11, Range = 68–100) compared with the control group ($\bar{X}$ = 81.56, SD = ± 7.01, Range = 66–100) (P = 0.023); however, our calculation yielded the level of acceptable rankings for both arms of the study.

**Table 3:** The mean value scores and standard deviations for the dimensions for Course Experience Questionnaire (CEQ), Nursing Students’ Satisfaction Scale (NSSS), Competency Inventory of Nursing Students (CINS) and Comprehensive Theoretical Knowledge and an Objective Structured Clinical Examination (OSCE).

Scales	Control Arm (n = 32)			Intervention Arm (n = 34)			P
	$\bar{X}$	SD	Category	$\bar{X}$	SD	Category	
**CEQ**	**110.41**	**11.28**	**Moderate**	**125.33**	**19.30**	**High**	**0.0001**
Good teaching	25.41	3.09		28.14	4.77		
Clear goal & standards	14.48	2.60		16.79	2.97		
Generic skills	18.67	2.98		21.64	4.36		
Appropriate assessment	17.87	2.78		20.66	3.28		
Appropriate workload	15.19	2.58		17.06	2.88		
Emphasis on independence	18.77	2.04		21.02	3.77		
**NSSS**	**109.76**	**16.94**	**High**	**117.87**	**15.94**	**High**	**0.049**
Curriculum & teaching	55.05	7.57		58.40	7.76		
Environment	18.55	3.89		19.59	4.75		
Professional social interaction	36.14	6.81		39.87	5.93		
**CINS**	**222.11**	**39.94**	**High**	**238.70**	**23.07**	**High**	**0.04**
Ethics & accountability	75.70	15.46		89.14	9.30		
General clinical skills	35.52	5.64		37.08	5.54		
Lifelong learning	30.64	7.50		33.94	3.91		
Clinical biomedical science	24.32	3.37		26.08	3.44		
Caring	31.94	5.99		32.44	4.02		
Critical thinking & reasoning	19.67	3.67		20.00	2.54		
**OSCE**	**81.56**	**7.01**	**Acceptable**	**85.40**	**6.11**	**Acceptable**	**0.023**

## Discussion

We implemented our pilot study with the objective of assessing the potential applicability and acceptance of OBE pedagogical technique by the “Gen Z” students in the field of maternal and pediatric nursing healthcare. Students in the intervention arm of the study scored higher in all the educational categories (nursing competencies, course experience, course satisfaction and assessment of theoretical knowledge and clinical proficiency). Our findings concur with previous studies suggesting the superiority of the OBE pedagogical technique over the traditional nursing didactic technique in effectiveness of dissemination of information and overall students’ satisfaction with their practicum [18, 19, 28–30]. Findings from our pilot study suggest that the OBE pedagogical technique is a preferred didactic method by our students. Furthermore, results from our current study suggest that the OBE pedagogical technique is superior to the traditional didactic technique in disseminating the clinical application of the pediatric nursing practicum.

Findings from our pilot study indicated a significant improvement in competency in clinical performance and assessment of theoretical knowledge of students with OBE pedagogy compared with the traditional didactic approach. Our findings concur with previously reported studies by Valizadeh et al. (2009), Nadery et al. (2012) and Fan et al. (2015) [28–30]. Current research supports that under the auspice of OBE pedagogical technique, learners are more motivated and encouraged to perform at a higher level in health data collection, physical assessment, and scenario simulation [19].

We set forth that the OBE pedagogical technique, which is based on the advancements in cognitive psychology, encourages students to become independent learners and active seekers of skill acquisition [31]. Additionally, we argue that the OBE pedagogical technique incentivizes students to improve their skills performance; this improvement can be demonstrated by higher final grades in their clinical practicum [30]. Results from our pilot study support that the OBE pedagogical technique facilitates students’ understanding of the clinical applications of their nursing practicum.

Nursing professionals in Mongolia carry the essential roles and responsibilities of delivery of maternal and pediatric clinical services, especially in remote regions of the country. Improving clinical dexterity of nursing students in Mongolia has become a priority for the Mongolian Ministry of Health and Social Services [32]. In response, academia in Mongolia has assumed several measures to strengthen nursing clinical didactics. These didactic approaches are effective only if students are mentally engaged with their education and learnings. The overarching goal of MNUMS is to prepare the future generations of nursing professionals with adequate clinical proficiencies to effectively address the national objective of reducing the gap in maternal mortality between urban and rural areas. The nursing professionals in Mongolia are the essential and frontline healthcare providers in providing pre- and/or post-natal healthcare services, particularly in remote *aimags* (provinces). Findings from our present pilot project will be applied toward larger future studies to assess the feasibility of OBE pedagogical technique as a nation-wide academic policy. Our long-term goal is to elevate the nursing education and clinical training for nursing students in Mongolia to the level of excellence.

### Limitations

Our study has several limitations. First, the semi quasi-experimental study did not allow for randomization of the study participants. Second, the study was implemented in only one school of nursing, MNUMS, owned and operated by the Mongolian government. Therefore, its applicability might be limited to other settings such as private universities. Meanwhile, findings from this pilot study have shed light on the applicability of OBE in academic nursing in Mongolia and has paved the road for implementation of a larger scale study.

### Implications

OBE pedagogical technique in nursing education could be of great benefit to improve nursing students’ competencies and higher learning satisfaction in provision of high-quality education. Nurse educators need to work in tandem to reach a consensus for developing strategies in overcoming barriers to implement OBE pedagogical approaches.

### Conclusion

OBE pedagogical approaches in nursing education could be of great benefit to improving nursing students’ competencies; furthermore, OBE offers a higher learning satisfaction and provision of high-quality education for the students. The preliminary findings from our pilot study shed light on the utility of novel and effective approaches in academic nursing. Novel and state of the art teaching approaches should be adopted and implemented to nourish critical thinking and clinical dexterity of nursing students.
